# Multi-Channel Exploration of O Adatom on TiO_2_(110) Surface by Scanning Probe Microscopy

**DOI:** 10.3390/nano10081506

**Published:** 2020-07-31

**Authors:** Huan Fei Wen, Yasuhiro Sugawara, Yan Jun Li

**Affiliations:** 1 Key Laboratory of Instrumentation Science and Dynamic Measurement, School of Instrument and Electronics, North University of China, Taiyuan 030051, China; wenhuanfei@nuc.edu.cn (H.F.W.); sugawara@ap.eng.osaka-u.ac.jp (Y.S.); 2 Department of Applied Physics, Graduate School of Engineering, Osaka University, 2-1 Yamadaoka, Suita, Osaka 565-0871, Japan

**Keywords:** multi-channel, surface property, scanning probe microscopy

## Abstract

We studied the O_2_ dissociated state under the different O_2_ exposed temperatures with atomic resolution by scanning probe microscopy (SPM) and imaged the O adatom by simultaneous atomic force microscopy (AFM)/scanning tunneling microscopy (STM). The effect of AFM operation mode on O adatom contrast was investigated, and the interaction of O adatom and the subsurface defect was observed by AFM/STM. Multi-channel exploration was performed to investigate the charge transfer between the adsorbed O and the TiO_2_(110) by obtaining the frequency shift, tunneling current and local contact potential difference at an atomic scale. The tunneling current image showed the difference of the tunneling possibility on the single O adatom and paired O adatoms, and the local contact potential difference distribution of the O-TiO_2_(110) surface institutively revealed the charge transfer from TiO_2_(110) surface to O adatom. The experimental results are expected to be helpful in investigating surface/interface properties by SPM.

## 1. Introduction

Scanning probe microscopy (SPM) has developed as a powerful tool for exploring the surface properties and surface dynamic process at an atomic scale on a semiconductor or insulator [1,2,3,4,5,6,7,8,9,10]. For example, atomic manipulation has been realized, and surface chemical reactions have been observed with atomic resolution by atomic force microscopy (AFM). Based on AFM, Kelvin probe force microscopy (KPFM) was developed to characterize the contact potential difference (CPD) between the substrate and cantilever tip. CPD originates from the difference of the work functions and is specifically referred to as the local CPD (LCPD) in atomic-resolution KPFM [11,12]. To date, different modes of KPFM have been successfully used to simultaneously measure surface structures and LCPD, and the surface potential of TiO_2_(110) was measured [13,14,15,16,17,18,19]. Local density of states (LDOS) gives significant information of the electronic structure of the surface, which is measured by scanning tunneling microscopy (STM). Combining AFM/STM techniques has been developed to investigate the surface structure and LDOS [20,21,22]. Simultaneous measurement of tunneling current and LCPD is useful to explore the surface properties and surface reaction process, but it cannot be achieved by the conventional KPFM due to the regulation of DC bias voltage. To simultaneously characterize the electronic structure and LCPD, we previously proposed a method to achieve the frequency shift (∆*f*), average tunneling current (<*I*_t_>) and LCPD by the KPFM without DC bias voltage feedback, and this method was successfully performed on the rutile TiO_2_(110) surface [19]. Hence, based on AFM, multi-channel exploration has the potential to explore the charge transfer of the interface and to unravel physical features for understanding the surface catalytic process.

Oxygen molecular interaction with TiO_2_ has attracted much attention and has become a prototypical model due to its importance in many surface reactions. Molecular oxygen acts as the main oxidizing reagent in many catalytic reactions and is used as an electron scavenger, which is believed to facilitate surface reactions. The adsorption and dissociation of O_2_ on the rutile TiO_2_(110) surface at low and room temperatures have been well characterized by STM experimental and theoretical study, where the oxygen molecular adsorbed on the Ti_5c_ site or the surface bridging oxygen vacancies (O_v_) and the oxygen adatom (O_ad_) along the fivefold-coordinated Ti_5c_ site is formed [23,24]. Importantly, it is believed that chemical adsorption and dissociation of O_2_ on the TiO_2_ surface accompanied by the charge transfer and the activation of O_2_ dissociation are key factors in the reaction process, which is importantly related to their charge state. Therefore, multi-channel exploring of the state of O_ad_ on TiO_2_(110) surface is useful in understanding its fundamental mechanism.

In this study, we first showed the dissociation state of O_ad_ on the TiO_2_(110) surface under the different O_2_-exposed temperatures by AFM, and then we characterized the O_ad_ by simultaneous AFM/STM, which would provide complementary information in recording the topographic and tunneling current signals. Finally, simultaneous measurements of frequency shift, tunneling current and LCPD images on the O-TiO_2_(110) surface are taken by SPM. The tunneling current contrast and surface potential difference were analyzed to explore the charge transfer between the O_ad_ and TiO_2_ surface. The experimental results and methods are useful in characterizing the other nanomaterials.

## 2. Experimental Details

**Figure 1:**
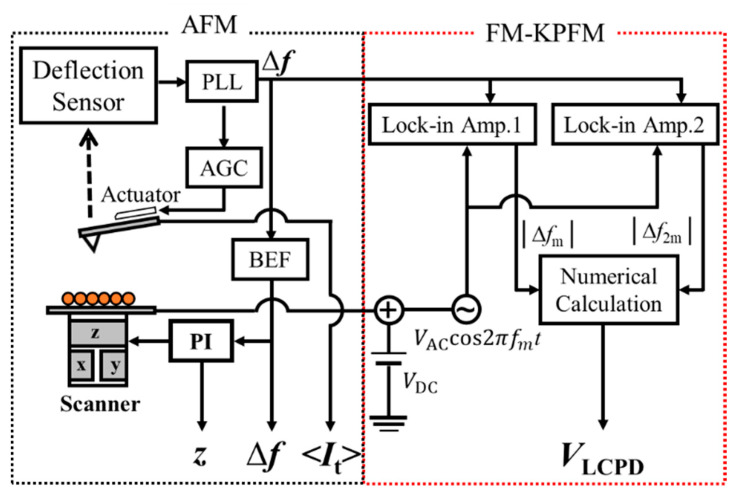
Experimental setup of AFM/FM-KPFM for simultaneous measurements of topography, <*I*_t_> and LCPD.

Experiments were performed with a home-built no contact (NC)-AFM system under ultrahigh vacuum conditions (3 × 10^−11^ Torr) at 78 K, which was operated in frequency modulation (FM). An AFM cantilever was oscillated at a constant amplitude and at its resonant frequency by automatic gain control (AGC). AFM/STM simultaneous measurements can be carried out in two ways, and force signal and tunneling current are recorded in the separated channel. When the frequency shift was operated as the feedback signal, topographic and <*I*_t_> images were simultaneously recorded and <*I*_t_> contained crosstalk of tip motion. When measurements were taken in the constant height mode, frequency shift and <*I*_t_> could be simultaneously obtained. In this mode, two signals were independently measured, so they did not contain the artificial signal. The comparison of two operation modes will be shown in the results section. Figure 1 shows the experimental setup for simultaneous measurements of topography, <*I*_t_> and LCPD. Topography and <*I*_t_> images were obtained by AFM, and LCPD images were recorded by FM-KPFM in the constant height mode. The equation for *V*_LCPD_ was derived as follows; details can be found in Ref. [18,19]:

(1) $$V_{\mathrm{LCPD}}=V_{\mathrm{DC}}-\mathrm{sgn}(\alpha_{m})\frac{V_{\mathrm{ac}}}{4}\frac{\left|\Delta f_{m}\right|}{\left|\Delta f_{2m}\right|}\frac{T(f_{2}{}_{m})}{T(f_{m})}$$

Here, *V*_DC_ is the dc voltage. The parameter sgn(α_m_) is known by the phase difference between *V*_ac_ and |∆*f*_m_|. |∆*f*_m_| and |∆*f*_2m_| are the *f*_m_ and *f*_2m_ components of ∆*f*, respectively. The *T*(*f*_m_) and *T*(*f*_2m_) are the transfer functions of the phase-locked-loop (PLL).

The signal frequency shift was obtained by the phase locked loop (PLL) and divided into two parts. One was applied to adjust the tip–sample interaction with a band elimination filter (BEF), and the other was connected to FM-KPFM by feeding it into the lock-in amplifiers. An ac bias voltage was obtained by an oscillator and acted as the reference signal. The *f*_m_ and *f*_2m_ components of the frequency shift were detected by two lock-in amplifiers. As shown in Figure 1, <*I*_t_> was recorded in a separate channel from the tip to cancel the crosstalk, and bias voltage was applied to the sample.

The commercial Ir-coated cantilever (Nanosensors SD-T10L100, *f*_0_ ~800 kHz) was used in the current study. The cantilever tip was first degassed at approximately 650 K for 30 min and then cleaned by Ar ion bombardment to remove the contaminants, prior to the measurements. Features of the surface structure were related to the charge states of the tip apex, and a stable tip was essential to accurately characterize the surface structure and properties in the experiment [25,26]. The imaging mode became stable in AFM experiments when the metal-coated Si cantilever was employed in the experiments [27,28,29].

The TiO_2_(110) sample surface (provided by Furuuchi Chemical Corporation, Hyogo, Japan) was prepared by several cycles of Ar ion sputtering and subsequent annealing at 1000 K for 20 min. After that, the freshly cleaned surface was exposed to O_2_ in the preparation chamber and then transferred into the observation chamber. AFM images were taken after the sample temperature decreased to 78 K.

## 3. Results and Discussion

**Figure 2:**
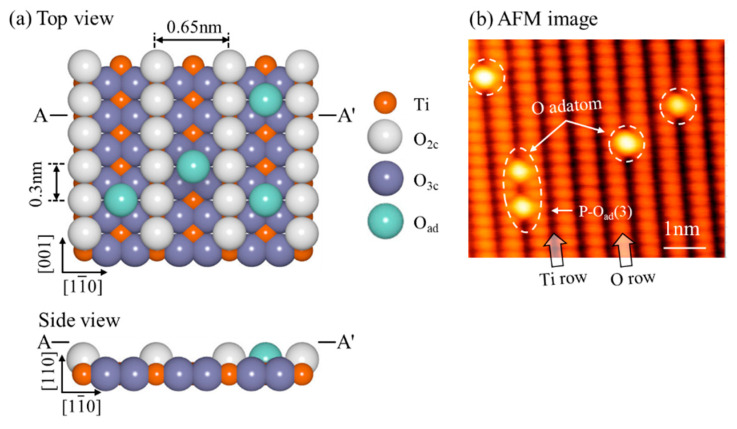
(**a**) Ball model of the rutile O-TiO_2_(110)-(1 × 1) surface. (Ti_5c_: orange balls, O_3c_: dark blue balls, O_2c_: gray balls, O_ad_: light green balls) (**b**) AFM image of O-TiO_2_(110)-(1 × 1) surface. Bright and dark rows are O_2c_ and Ti_5c_ rows, respectively, and bright spot is O_ad_. (*f*_0_ = 807 kHz, *Q* = 23620, ∆*f* = −70 Hz, *V*_DC_ = 0.6 V and *A* = 500 pm, image size: 6.3 × 5.3 nm^2^).

We first introduced the surface structure model of rutile O-TiO_2_ (110)-(1 × 1) and O_2_ dissociation state at room temperature (RT) and 400 K. Figure 2a shows a ball model of the rutile O-TiO_2_ (110)-(1 × 1) surface, which consists of alternating Ti_5c_ rows and sixfold-coordinated Ti_6c_ rows surrounded by in-plane threefold-coordinated O_3c_ rows and bridging twofold-coordinated O_2c_ rows. The single O_ad_ (O_ad_: light green ball) formed by O_2_ dissociation at the O_v_ site indicated that one O atom healed O_v_ and the other located at the Ti_5c_ site, and paired O_ad_ resulted from O_2_ dissociation at the Ti_5c_ site.

As reported in the previous literature [30,31,32], atomic contrast in the AFM image depended on the tip apex polarity, and surface defects were used as markers to distinguish the imaging mode. Hole and protrusion modes usually appeared in the imaging modes. When the tip apex was positively charged, the O_2c_ row was bright on the image due to the larger attractive force between the tip and the negative O_2c_ row. Surface defects were imaged as dark holes, which is called the hole mode [28,29]. When the tip apex was negatively charged, the contrast was inverted compared with that in the hole mode, and H atoms appeared as brighter spots than the O_V_ defects, which is called the protrusion mode. Figure 2b shows the topography image of the O-TiO_2_(110)-(1 × 1) surface recorded in the hole mode. According to the experiment, bridging O_2c_ and Ti_5c_ rows were imaged as bright and dark features, respectively, and the bright spots on the Ti_5c_ rows are O_ad_. As introduced before, the single O_ad_ (denoted by the white dotted circle) was attributed to O_2_ dissociation at the O_v_ site, and paired O_ad_ separated by three lattice distances (denoted by the white dotted oval circle) was the result of O_2_ dissociation at the Ti_5c_ site.

**Figure 3:**
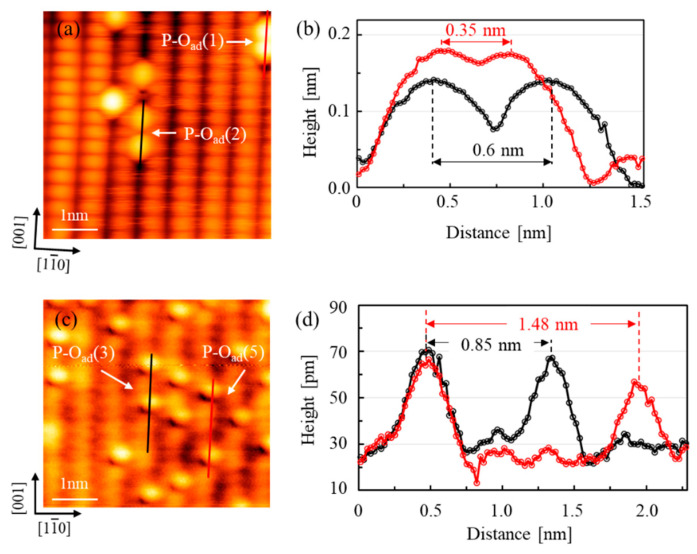
(**a**) AFM images on TiO_2_(110) surface. (*f_0_* = 802 kHz, *Q* = 13001, ∆*f* = −80 Hz, *V*_DC_ = 0.4 V, *A* = 500 pm, 5 × 5 nm^2^, O_2_ exposure at RT). (**b**) Line profiles along the oxygen adatoms in (**a**). (**c**) AFM images on TiO_2_(110) surface. (*f_0_* = 795 kHz, *Q* = 6329, ∆*f* = −231 Hz, *V*_DC_ = 0.4 V, *A* = 500 pm, 4.5 × 5 nm^2^, O_2_ exposure at 400K). (**d**) Line profiles along the oxygen adatoms in (**c**).

Figure 3 shows two AFM topographic images of the rutile O-TiO_2_(110)-(1 × 1) surface exposed to O_2_ at RT and 400 K, respectively, and the corresponding line profiles along the O_ad_. The contrast is the same as Figure 2b. O_2c_ and Ti_5c_ rows are imaged as bright and dark features, respectively, and the bright spots on the Ti_5c_ rows are O_ad_. Here, paired O_ad_ separated by one lattice constant is denoted as the P-O_ad_(1). As shown in Figure 3a,b and Figure 2b, single O_ad_, P-O_ad_(1), P-O_ad_(2) and P-O_ad_(3) are observed when O_2_ is exposed to the TiO_2_ surface at room temperature (RT). As introduced before, single O_ad_ was attributed to O_2_ dissociation at the O_v_ site, and paired O_ad_ was the result of O_2_ dissociation at the Ti_5c_ site. In our results, a single O adatom was the distinctly dominant state of O_ad_, when the exposure temperature was at RT. P-O_ad_(2) is the second preferred state. Density functional theory (DFT) showed the P-O_ad_(2) configuration was the most preferred structure at RT, and further O_ad_ diffusion (P-O_ad_(2) to P-O_ad_(3)) was hindered by a barrier of 1.3 eV [24]. In addition, the separation of P-O_ad_(1) to P-O_ad_(2) was exothermic by 0.4 eV theoretically. P-O_ad_(1) and P-O_ad_(3) configurations were observed in our experiments, but they were rare because separation of O_ad_ is the result of a balance between Coulombic repulsion of two O_ad_ and is thermally driven, and P-O_ad_(1) and P-O_ad_(3) configurations can be generated.

When the sample was exposed to O_2_ conditions beyond 350 K, dissociated O_2_ could overcome the diffusion barrier forming the P-O_ad_(3) structure [33]. The number of paired O_ad_ distinctly increased on the O_2_-exposed surface at 400 K, and the states of the paired O_ad_ were mainly P-O_ad_(3) and P-O_ad_(5) configurations, shown in Figure 3c,d. Under high temperatures for the O_2_-exposed surface (>400 K), the Ti interstitials (Ti_int_) can diffuse from the bulk to the near-surface region via an interstitial diffusion mechanism [34]. The concentration and distribution of Ti_int_ in the near-surface region can vary significantly, depending on the level of bulk reduction. Current experiments suggest that the excess charge on these paired O_ad_ is mainly provided by the Ti_int_ rather than Ov in determining the adsorption behavior when the O_2_-exposed surface temperatures went beyond 400 K. Our results demonstrate that O_2_ dissociatively adsorbed on the rutile TiO_2_(110)-(1 × 1) surface when the temperature of O_2_ exposure was at or beyond RT, and results are consistent with the conventional STM observations [24,33]. Next, we explored the O_ad_ on TiO_2_ surface by AFM/STM.

**Figure 4:**
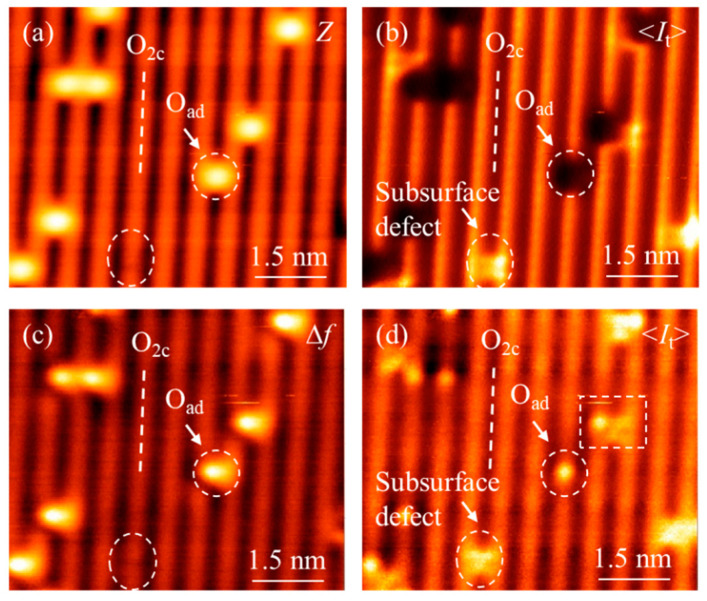
(**a**,**b**) Topographic and corresponding tunneling current images of O-TiO_2_(110) surface obtained in the constant frequency shift mode. There is crosstalk between topography and <*I*_t_> signals and O_ad_ is imaged dark spot in <*I*_t_> image. (**c**,**d**) frequency shift and corresponding tunneling current images of O-TiO_2_(110) surface obtained in the constant height mode. The crosstalk between topography and <*I*_t_> signals is eliminated.

The four images in Figure 4 are obtained in the same area of the O-TiO_2_(110) surface. Figure 4a and 4b experimentally show simultaneously recorded topographic (*Z*) and <*I*_t_> images recorded in the constant frequency shift mode. In the topographic image (Figure 4a), the atomic contrast is the same as that in Figure 3a, in that the bright and dark rows are the O_2c_ and Ti_5c_ rows, respectively. A bright spot marked by the dashed white circle is O_ad_. Figure 4b demonstrates the corresponding tunneling current image. Usually, the empty state is imaged at positive sample bias voltage in STM, so O_2c_ and Ti_5c_ rows are imaged as dark and bright rows, respectively, where the contrast of O_2c_ and Ti_5c_ rows is reversed compared with the topographic image. The dark spot marked by the white dashed circle is O_ad_, and this is different from the conventional STM image of O-TiO_2_(110), which is caused by the crosstalk of tip motion. When the tip moves on the O_ad_ site, the additional attractive force acts in the tip–sample interaction, and the tip has to retract in order to keep a constant frequency shift. Thus, tunneling current dramatically decreases, and the contrast of O_ad_ becomes a dark spot in <*I*_t_> image. The bright spot marked by an oval circle is due to subsurface defects, which was not probed in Figure 4a, ever reported by our group or other groups [19,22]. Figure 4c is the ∆*f* image and Figure 4d is the corresponding tunneling current image, recorded in the constant height mode. Compared with Figure 4a,b, the image contrast is the same, except the O_ad_ in Figure 4d. O_ad_ is imaged as bright spot in the <*I*_t_> image due to elimination of the crosstalk between topography and <*I*_t_> signals in the constant height mode. A bright spot and two weak bright spots (denoted by the white square) are observed in Figure 4d, and they are O_ad_ and subsurface defects. It indicates the subsurface defect is not repulsive to O_ad_. Therefore, AFM/STM is a useful technique to investigate the interaction of the adsorbate and substrate, and constant height operation is necessary. Next, we investigated the O_ad_ by AFM/STM/KPFM.

**Figure 5:**
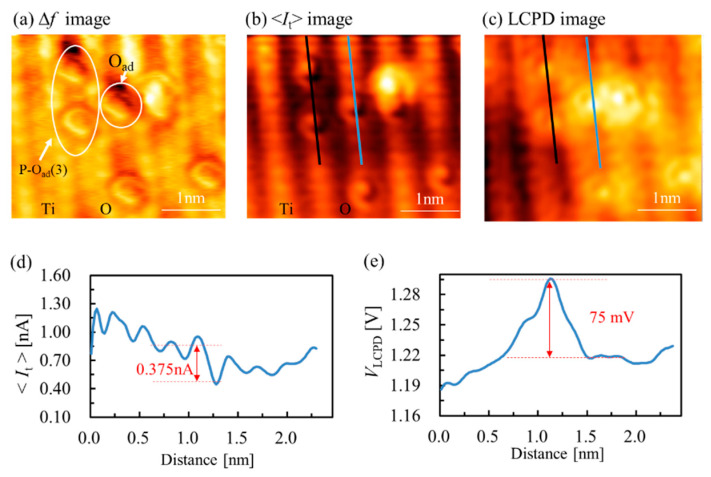
Multiple images of TiO_2_(110) surface with atomic resolution and corresponding line profiles. (**a**) Frequency shift (∆*f*) image, (**b**) tunneling current (<*I*_t_>) image and (**c**) local contact potential difference (*V*_LCPD_) image. (**d**,**e**) The line profiles along the blue line on the surface in (**b**,**c**). (*f*_0_ = 805 kHz, *Q* = 27623, ∆*f* = −260 Hz, *V*_DC_ = 1.3 V, *V*_AC_ = 1.5 V, *A* = 500 pm, size: 3.5 × 3.2 nm^2^).

Figure 5 shows frequency shift, tunneling current and local contact potential difference images with atomic resolution and corresponding line profiles along the single O_ad_. In the experiment, the measurement was performed in the constant height mode to prevent crosstalk between the signals of the frequency shift and tunneling current. In the *Δf* image shown in Figure 5a, O_2c_ and Ti_5c_ rows are simultaneously observed as bright rows with super high resolution, and O_ad_ is imaged as the bright spot. In the <*I*_t_> image (see Figure 5b), the contrast was the same as in Figure 5a, except O rows are imaged as dark, which is consistent with the previous studies by conventional STM in that the conduction band of TiO_2_ is dominated by Ti 3*d* states, and bright features are usually assigned to the empty Ti 3*d* states of Ti_5c_ ions under positive sample bias voltage, even though they lie lower than the bridging O_2c_ rows [35]. The tunneling current value on O_ad_ was higher than that on the Ti_5c_ rows, and the current difference between the O_ad_ and Ti_5c_ row was about 0.375 nA. The contrast in STM images depends on the different contributions of Ti 3*d* and O 2*p* states and their different decay as a function of the tip–sample separation [36]. O_ad_ is higher than O_2c_ and Ti_5c_ rows in surface geometry, and O_ad_ finally appeared as bright on the image. It was clearly observed that there was some depression around the O_ad_, and this was due to the decreased numbers of empty states near the conduction band for tunneling, which resulted from the negatively charged O_ad_ [24]. This phenomenon was more pronounced in paired O_ad_, as shown in the following tunneling current line profile. In the *V*_LCPD_ image (see Figure 5c), the imaging contrast was the reverse of that in the image, except for the O_ad_. The relative value of *V*_LCPD_ between the Ti_5c_ and O_2c_ sites was approximately 28 mV from the line profile (not shown here), and this was in good agreement with our previous studies [18,19]. *V*_LCPD_ had a higher value on the O_ad_ than on the proximate Ti_5c_ rows, and the relative value of CPD between the O_ad_ and Ti_5c_ row was about 75 mV. It intuitively suggests the electrons transferred from TiO_2_ to O_ad_.

**Figure 6:**
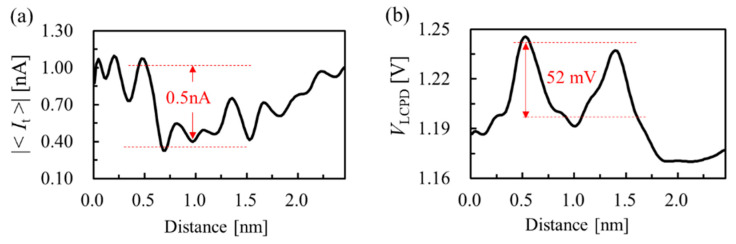
The line profiles along the dark line on the surface in Figure 5b,c. (**a**) Tunneling current and (**b**) local contact potential difference line profiles along the paired O adatoms.

As shown in Figure 6, line profiles are plotted along the paired O_ad_. The current difference between the O_ad_ and adjacent Ti_5c_ row was about 0.5 nA. The depression between the paired O_ad_ was very pronounced due to the combined effect in the corresponding line profile. The surface potential distribution was also different around the single O adatom and paired O_ad_. The potential difference was 52 mV, as shown in Figure 6b. This indicates the charge state of O_ad_ is different, and excess electrons around the paired O_ad_ are shared by the paired O_ad_ resulting in that the relative value of CPD between the O_ad_ and Ti_5c_ row is lower. Therefore, multi-channel exploration demonstrates the powerful ability to explore surface properties with SPM.

## 4. Conclusions

We studied the O_2_ dissociated state under different O_2_-exposed sample surfaces with high resolution, and we investigated the electron charge transfer between the adsorbed O and TiO_2_ substrate with multi-channel exploration. We observed the interaction between O_ad_ and the subsurface, and we confirmed the electron charge transfer from the TiO_2_ surface to the adsorbed O upon O_2_ dissociation on the TiO_2_(110) surface by tunneling current and local contact potential difference. Our results demonstrated that multi-channel exploration was able to obtain the surface structures and charge transfers between the adsorbate and substrate, and this is expected to be useful for investigating the surface properties and charge transfer phenomena at the interface.
